# Investigating the Convergence between Actigraphy, Maternal Sleep Diaries, and the Child Behavior Checklist as Measures of Sleep in Toddlers

**DOI:** 10.3389/fpsyt.2014.00158

**Published:** 2014-11-10

**Authors:** Marie-Ève Bélanger, Valérie Simard, Annie Bernier, Julie Carrier

**Affiliations:** ^1^Department of Psychology, University of Montreal, Montreal, QC, Canada; ^2^Department of Psychology, University of Sherbrooke, Sherbrooke, QC, Canada; ^3^Center of Advanced Research in Sleep Medicine, Hôpital du Sacré-Coeur de Montréal, Montreal, QC, Canada

**Keywords:** actigraphy, sleep diary, CBCL, sleep, toddlers, agreement

## Abstract

The current study examined associations among actigraphy, maternal sleep diaries, and the parent-completed child behavior checklist (CBCL) sleep items. These items are often used as a sleep measure despite their unclear validity with young children. Eighty middle class families (39 girls) drawn from a community sample participated. Children (*M* = 25.34 months, SD = 1.04) wore an actigraph monitor (Mini-Mitter^®^ Actiwatch Actigraph, Respironics) for a 72-h period, and mothers completed a sleep diary during the same period. Eighty-nine percent of the mothers and 75% of the fathers also filled out the CBCL (1.5–5). Mother and father CBCL scores were highly correlated. Overall, good correspondence was found between the CBCL filled out by mothers and sleep efficiency and duration derived from maternal sleep diaries (*r* between −0.39 and −0.25, *p* ≤ 0.05). Good correspondence was also found between the CBCL filled out by fathers and sleep efficiency as derived from maternal sleep diaries (*r* between −0.39 and −0.24, *p* ≤ 0.05), but not with sleep duration (all results were non-significant). Very few correlations between actigraphy and the CLBL scores reached statistical significance. The Bland and Altman method revealed that sleep diaries and actigraphy showed poor agreement with one another when assessing sleep duration and sleep efficiency. However, diary- and actigraphy-derived sleep durations were significantly correlated. Consistent with findings among older groups of children, this study suggests that the CBCL sleep items, sleep diaries, and actigraphy tap into quite different aspects of sleep among toddlers. The choice of which measures to use should be based on the exact aspects of sleep that one aims to assess. Overall, despite its frequent use, the composite sleep score of the CBCL shows poor links to objective measures of sleep duration and sleep efficiency.

## Introduction

Studies estimate that between 10 and 75% of parents report that their children have sleep problems (1). In light of the prevalence and the serious consequences of pediatric sleep difficulties for behavioral, cognitive, and emotional health (2–6), it is essential to accurately measure sleep quality in young children in both clinical and research contexts.

Different instruments are used to assess sleep in young children [e.g., polysomnography (PSG), actigraphy, prospective sleep diaries, and retrospective questionnaires]. Each of these measures has its advantages and disadvantages (7). The child behavior checklist [CBCL; (8)], which contains items that retrospectively assess specific sleep problems, is widely used by clinicians and researchers, although its primary focus is not the assessment of sleep (9). The CBCL has the twofold advantage of being cost-effective and of focusing specifically on sleep complaints, which is useful in clinical settings. Studies indicate that the CBCL sleep problems scale is able to discriminate between snoring and non-snoring preschoolers (10), as well as between typically developing toddlers and those diagnosed with Williams syndrome (11). However, retrospective parental questionnaires like the CBCL are, in general, quite susceptible to respondent biases. Sleep diaries are widely used in sleep research with infants and children (7). The diary records, on a timeline of 24 h, the sleep–wake pattern of the child as it progresses. Thus, sleep diaries, while also cost-effective, provide a prospective and quantitative measurement of sleep duration and sleep–wake schedule. However, like retrospective child sleep questionnaires, prospective sleep diaries are often criticized for their reliance on parental awareness of child sleep (e.g., nocturnal awakenings), which itself can depend on the child’s propensity to signal his or her awakenings or difficulty falling asleep. In contrast, actigraphy is an objective sleep measure, which uses a watch-size movement sensor to determine sleep and wake episodes. It is non-invasive and allows for multiple-day data collection in the child’s natural environments (e.g., home, daycare, and school), thereby conferring ecological validity to collected sleep data. However, its ability to detect wakefulness in young children is poor (12, 13), and movement artifacts (e.g., a child sleeping in a moving car or stroller), which are a potential source of error, constitute a limitation of this sleep measure.

Many sleep scholars have examined the degree of correspondence between different types of sleep measures in infants (14, 15) and among preschool- and school-aged children (16–19). The literature to date suggests that when sleep quality variables (e.g., percentage of time spent asleep during the sleep period or sleep efficiency) are considered, the correspondence between actigraphy and subjective reports is relatively poor, yet when sleep schedule variables are considered (e.g., sleep onset, sleep offset, sleep duration), correspondence is higher. However, to our knowledge, research has yet to estimate the extent to which different sleep assessment methods converge when they are used with toddlers. Also, most of the studies that have examined between-methods correspondence have done so through correlations or between-group comparisons, which, as pointed out by Werner et al. (20), limit our understanding of how equivalent or interchangeable the methods are. Furthermore, only one study has examined the correspondence between the CBCL sleep items and other sleep measures. Studying clinical and non-clinical samples of children aged 7 through 17 years old, Gregory et al. (9) observed that many of the expected associations between the CBCL and objective sleep measures were not found. Nonetheless, studies using the CBCL sleep items with toddlers or preschoolers often refer to the Gregory et al. (9) study to support their methodological choice, failing to note that this study was conducted with older children and adolescents and, in fact, revealed modest concordance with objective sleep measures (actigraphy and PSG). Moreover, the Gregory et al. study used the 4–18-year version of the CBCL (21, 22) as opposed to the 1.5–5-year version (8), which is usually used with toddlers and in which there are several items that differ from the 4–18-year version. Overall, the widespread utilization of the CBCL to assess sleep in toddlers and preschoolers stands in sharp contrast to the very scant evidence supporting its convergent validity.

Given the rise in sleep research with toddlers and preschoolers in recent years [e.g., Ref. (4, 23–26)] and the growing research pertaining to fathers’ roles in their children’s sleep [e.g., Ref. (6, 27–29)], it is critical that the field be clear on the pros and cons of different sleep measures with this age group, including maternal and paternal reports. Taking initial steps in this direction, the current study aims to examine the associations among actigraphy, a maternal sleep diary, and the CBCL sleep items completed by both parents of typically developing toddlers. This study is mainly exploratory; nevertheless, it was expected that high correspondence would be found between the CBCL filled by the two parents, given that high cross-informant correlations have been reported between parents on this version of the CBCL (8). Moreover, moderate to high correspondence was expected between the CBCL filled by mothers and maternal diaries, given the single informant. Also, despite the low correspondence previously found with older children [e.g., Ref. (18, 19)], moderate correspondence between actigraphy and the diary was anticipated, given toddlers’ relatively high dependence on their caregivers for sleep regulation (in contrast to older children who may well remain awake for long periods at night and not call for their parents). Finally, in line with the results of Gregory et al. (9), poor relations were expected between the CBCL completed by either parent and actigraphy.

## Materials and Methods

### Participants

Eighty families (39 girls) living in a large Canadian metropolitan area participated in this study. Families were recruited from birth lists randomly generated and provided by the Quebec Ministry of Health and Social Services. Criteria for participation were full-term pregnancy and the absence of any known physical or mental disability. Families were assessed when children were 2 years old (*M* = 25.35 months, SD = 1.04). Most parents were Caucasian (93.8% of mothers, 83.8% of fathers). Mothers were between 20 and 44 years old (*M* = 31.92), and fathers between 21 and 47 years old (*M* = 33.99). Mothers had 16.0 years of education on average, ranging between 8 and 18 years, and 68.8% held a college degree. Fathers had 15.9 years of education on average, ranging between 11 and 21 years, and 68.8% held a college degree. Family income was based on categorical scores distributed as follows: 1 (*n* = 3) < 20k$; 2 (*n* = 7) = 20–39k$; 3 (*n* = 11) = 40–59k$; 4 (*n* = 23) = 60–79k$; 5 (*n* = 8) = 80–99k$; 6 (*n* = 28) = 99k$; and over. Mean family income for the sample was 4.41 (SD = 1.54), which was comparable to the $74,600 mean family income in Canada during the years of data collection. In light of their intercorrelations (*r*’s from 0.54 to 0.59), maternal and paternal education and family income were standardized and averaged into a global index of family SES.

### Procedure

Children wore an actigraph monitor for 72 h. Mothers were instructed to complete a diary of their child’s sleep during the same period. In addition, both parents were asked to complete the CBCL to assess the children’s sleep problem symptoms at home and then to return it by mail. Parents were invited to fill out the questionnaires independently and were each provided with a pre-paid envelope. Eighty-nine percent of the mothers and 75% of the fathers returned the questionnaire. Families in which parents did not complete the CBCL did not differ from others on socio-demographics or on child sleep as derived by actigraphy or diary (all *t*’s < 1.78, ns). The University’s Ethics Committee approved the research project. The parents of all participating children signed a consent form at the outset of the study that informed them of the nature and risks of participating, and they received financial compensation along with a toy for the child.

### Measures

#### Actigraphy

Children wore an actigraph monitor (Mini-Mitter^®^ Actiwatch Actigraph, Respironics) for 72 h. This brand of actigraphy, relative to PSG, has been reported to overestimate night awakenings in young children, thereby underestimating sleep time [e.g., Ref. (13, 30)] due to young children’s increased motor activity during sleep (31). Consequently, actigraphic data were analyzed initially with the automated manufacturer’s scoring algorithm set at high sensitivity (more appropriate for young children’s motor activity). A secondary “smoothing” algorithm, developed specifically to address the problem of overestimation of night waking (32), was then applied to the nighttime data. This algorithm has been validated against videosomnography (32) and home-based PSG (12). Young children often feel uncomfortable wearing an actigraph on their wrist, particularly at night. Therefore, mothers were informed that the child could wear the actigraph either on the wrist or the ankle and were asked to report this information to the research assistant (82.5% of the children wore the actigraph on the ankle). Location of the actigraph does not influence the data among toddlers and preschoolers or their correspondence with PSG: this model of actigraphy shows good to high agreement (77–98% across parameters) with PSG for this age group, regardless of the location of the monitor (12).

Valid sleep data were available for three nights for 64 participants, two nights for 9 participants, and one night for 7 participants. Sleep data were missing because children refused to wear the actigraph for a second or third day or had to be discarded because the diary indicated that the child had been asleep in a moving object (car, stroller). There was no significant difference according to the number of nights with available actigraphic data (1, 2, or 3 nights) for sleep duration [*F*(2,77) = 0.36, *p* = 0.70] and sleep efficiency [*F*(2,77) = 0.31, *p* = 0.74]. All available data were, therefore, used for each child.

Actigraphy-derived sleep variables, averaged across nights of assessment, were as follows: sleep duration (number of minutes scored as sleep between sleep onset and offset) and sleep efficiency [number of minutes scored as sleep between sleep onset and offset/(number of minutes scored as sleep + number of minutes scored as wake between sleep onset and offset) × 100]. These two sleep variables were chosen based on their demonstrated correspondence to PSG estimates when using this model of actigraphy at the same developmental period (12). The determination of sleep onset and offset was based on visual examination of actigraphic data for each night, especially around the time of sleep onset and offset as reported by the mother in the diary.

#### Sleep diary

Mothers were instructed to complete a sleep diary for the hours during which their child was wearing the actigraph. They were asked to indicate, for each half hour, whether the child was awake or asleep and where. Sleep duration at night and sleep efficiency were derived and averaged across the nights of assessment.

#### Child behavior checklist, 1.5–5-year version

Both parents completed the 100-item CBCL, 1.5–5-year version (8). The sleep problems scale that can be generated from the CBCL was used, summing up children’s scores on the seven sleep items (α = 0.78). The items are (1) “Does not want to sleep alone,” (2) “Has trouble getting to sleep,” (3) “Nightmares,” (4) “Resists going to bed at night,” (5) “Sleeps less than most children during day and/or night,” (6) “Talks or cries out in sleep,” and (7) “Wakes up often at night.” As with other items on the CBCL, parents were asked to describe their child’s behavior now or within the past 2 months on a 3-point Likert scale (0 = not true; 1 = somewhat or sometimes true; 2 = very true or often true). In line with Gregory et al. (9) and given the exploratory nature of the present study, analyses will consider each of the seven items separately, in addition to the sleep problems scale score.

### Plan of analysis

Partial correlations that controlled for confounding variables as well as the statistical approach proposed by Bland and Altman (33) were conducted to assess, respectively, degrees of relation and agreement rates between diary- and actigraphy-derived sleep estimates. Given that CBCL scores were not normally distributed, Spearman rank-order correlations were conducted to assess the degree of relation between both the diary- and actigraphy-derived sleep estimates and CBCL scores.

Preliminary analyses were conducted to identify potential confounds of sleep variables among biological (child gender, weeks of gestation, birth weight, duration of breastfeeding) and socio-demographic variables (birth order, parental work hours, ethnicity, SES). Only two of these variables were found to relate to sleep. SES was correlated with sleep variables estimated by mothers (diary and CBCL; *r* ≥ 0.18, *p* ≤ 0.033). Also, marginally higher sleep efficiency and longer sleep duration derived from the diary were found in girls when compared with boys [*t*(78) ≥ −1.832, *p* ≤ 0.071]. Accordingly, family SES and child sex were included as covariates in the partial correlation analyses.

Werner et al. (20) argue that only the Bland and Altman method is a suitable approach to examine the agreement between two measures since it provides an interval within which 95% of the differences between measures are expected to lie (limits of agreement). In contrast to correlations, this procedure does not focus on between-children differences but rather estimates the agreement between methods on a child-by-child basis. Bland–Altman limits of agreement are computed based on parameters (mean and SD) that characterize the distribution of the between-methods differences. Based on the size of the difference within which 95% of the cases lie, one can judge whether or not the between-methods agreement is satisfactory, given *a priori* defined thresholds. In the present study, satisfactory agreements were defined *a priori* as a between-methods difference of 90 min or less with respect to sleep duration and a difference of 10% or less with respect to sleep efficiency. These agreement criteria were chosen given the 30-min window of the diary and probable sleep latency (time elapsed from going to bed as marked on the diary to sleep onset as detected by actigraphy), sleep offset-getting up delay (time elapsed between sleep offset as detected by actigraphy and wake up time as marked on the diary), and nocturnal awakening (possibly unnoticed by the mother). In other words, the 90-min difference allows for gaps of about 30 min (one time window in the diary) at sleep onset, at sleep offset, and upon one nocturnal awakening, thereby representing a very lenient criterion. The 10% criterion with sleep efficiency was meant to parallel this leniency.

Given that the Bland and Altman procedure can only be used to examine between-methods differences on the same construct measured on the same scale (e.g., minutes, percentages, etc.), we could not use it to compare the CBCL to the other sleep measures. It was therefore used only to compare sleep diaries and actigraphy. As mentioned above, given that the distribution of all CBCL scores (seven items and the sleep problems scale) was positively skewed with this non-clinical sample, non-parametric analyses were conducted when this sleep measure was considered. Hence, Spearman rank-order correlations were performed to examine the associations between sleep problems as reported on the CBCL and other sleep measures (note, however, that these analyses cannot accommodate covariates).

## Results

Descriptive statistics for CBCL, diary, and actigraphy sleep variables are shown in Table 1.

**Table 1:** **Descriptive statistics for CBCL, diary, and actigraphy sleep variables**.

Sleep variables	*N*	Min	Max	Mean	SD
Actigraphy					
Sleep duration (min)	80	389.3	678.3	563.2	59.5
Sleep efficiency (%)	80	67.0	99.6	90.4	7.1
Diary					
Sleep duration (min)	80	487.5	772.5	638.7	48.4
Sleep efficiency (%)	80	81.8	100.0	98.3	3.7
CBCL sleep problems scale					
Mothers	71	0	10.0	3.1	2.7
Fathers	53	0	8.0	2.5	2.5

*Scores on the CBCL sleep problems scale can vary between 0 and 14, with higher scores representing more sleep problems*.

### CBCL filled out by mothers and fathers

Table 2 presents the Spearman rank-order correlations between maternal and paternal CBCL scores. All correlations between mothers’ and fathers’ scores were marginally or statistically significant.

**Table 2:** **Spearman rank-order correlations between CBCL maternal and paternal scores**.

	*r*
CBCL sleep problems scale	0.57**
Does not want to sleep alone	0.61**
Has trouble getting to sleep	0.26†
Nightmares	0.58**
Resists going to bed at night	0.27†
Sleeps less than most children during day and/or night	0.24†
Talks or cries out in sleep	0.46**
Wakes up often at night	0.31*

†p < 0.10; *p < 0.05; **p < 0.01.

### Sleep diary and CBCL

Table 3 presents the Spearman rank-order correlations between diary-derived sleep estimates and sleep problems as reported on the CBCL. Overall, all the statistically significant associations were in the expected direction (i.e., more severe sleep problems being correlated with shorter sleep duration or poorer sleep efficiency as assessed through the diary). However, the “Sleeps less than most children during day and/or night” item, assessed by either mother or father, was not correlated with either of the sleep variables derived from the diary. The CBCL sleep problems scale as rated by either mother or father was not associated with sleep duration derived from the diary but did relate to sleep efficiency. Finally, in contrast to sleep efficiency, sleep duration as assessed by the mother in the diary was not related to any sleep problems as reported by the father on the CBCL.

**Table 3:** **Spearman rank-order correlations between CBCL and sleep diary**.

	Diary sleep duration	Diary sleep efficiency
**CBCL FILLED BY MOTHERS**		
Sleep problems scale	−0.21	−0.31*
Does not want to sleep alone	−0.36**	−0.25*
Has trouble getting to sleep	−0.39**	−0.13
Nightmares	0.06	−0.27*
Resists going to bed at night	−0.28*	−0.14
Sleeps less than most children during day and/or night	−0.10	−0.15
Talks or cries out in sleep	0.04	−0.21
Wakes up often at night	−0.09	−0.35**
**CBCL FILLED BY FATHERS**		
Sleep problems scale	0.08	−0.36**
Does not want to sleep alone	−0.26	−0.35*
Has trouble getting to sleep	−0.02	−0.15
Nightmares	0.17	−0.39**
Resists going to bed at night	0.08	0.02
Sleeps less than most children during day and/or night	−0.01	−0.08
Talks or cries out in sleep	0.23	−0.27
Wakes up often at night	0.05	−0.34*

*p < 0.05; **p < 0.01.

### Actigraphy and CBCL

Spearman rank-order correlations were performed to examine the relation between actigraphy-derived sleep estimates and sleep problems as reported on the CBCL (Table 4). Overall, only 2 of the 32 correlations that were run between actigraphy and the CBCL scores reached statistical significance at the 0.05 level, which suggests a pattern of essentially null findings; however, the two exceptions may be meaningful, as addressed in the discussion.

**Table 4:** **Spearman rank-order correlations between CBCL and actigraphy**.

	Actigraphy sleep duration	Actigraphy sleep efficiency
**CBCL FILLED BY MOTHERS**		
Sleep problems scale	−0.17	−0.21
Does not want to sleep alone	−0.09	−0.01
Has trouble getting to sleep	−0.34**	−0.18
Nightmares	0.06	−0.06
Resists going to bed at night	−0.24*	−0.12
Sleeps less than most children during day and/or night	−0.11	−0.10
Talks or cries out in sleep	−0.07	−0.18
Wakes up often at night	0.02	−0.15
**CBCL FILLED BY FATHERS**		
Sleep problems scale	0.06	−0.15
Does not want to sleep alone	0.04	0.07
Has trouble getting to sleep	0.08	−0.14
Nightmares	0.19	0.01
Resists going to bed at night	0.10	−0.02
Sleeps less than most children during day and/or night	0.05	0.01
Talks or cries out in sleep	−0.01	−0.18
Wakes up often at night	0.10	−0.03

*p < 0.05; **p < 0.01.

### Sleep diary and actigraphy

After controlling for child gender and SES, actigraphy- and diary-derived sleep duration estimates were positively correlated (*r* = 0.30, *p* = 0.007). However, the two measurement methods were uncorrelated when considering sleep efficiency (*r* = 0.02, ns).

The agreement between actigraphy and sleep diary was estimated using the Bland and Altman (33) method. First, analyses were conducted to check whether assumptions for calculating the 95% limits of agreement using a parametric approach were met (33). One basic assumption was violated: that is, a severe deviation to normality was observed in regards to the between-methods differences (i.e., the distributions were positively skewed). This was manifested in two threats to the validity of the parametric approach of the Bland and Altman method in the present dataset. First, the SD of between-methods differences for sleep duration was large, which would result in artificially inflated 95% limits of agreement. Second, there was a linear relation between average values and between-methods differences with respect to sleep efficiency, which represents a serious threat to a parametric Bland and Altman analysis (33). Following Bland and Altman’s recommendation, data were therefore log-transformed, but this procedure had little impact on the distributions. Accordingly, the Bland and Altman non-parametric approach to estimating between-methods agreement was preferred, as suggested and described by these authors (33). This non-parametric approach of the Bland and Altman method consists in reporting the proportion of cases falling into specified ranges of between-methods differences (see Table 5). Based on the *a priori* agreement criteria, agreement was satisfactory for 70.0% of children with respect to sleep duration and for 71.3% of children with respect to sleep efficiency.

**Table 5:** **Agreement between actigraphy and sleep diaries using the Bland and Altman non-parametric approach**.

**SLEEP VARIABLES**				
Sleep duration	≤30 min	≤60 min	≤90 min^a^	≤120 min
	25.0	46.2	70.0	78.7
Sleep efficiency	≤5%	≤10%^a^	≤15%	≤20%
	37.5	71.3	83.7	92.5

*^a^Satisfactory agreement criteria*.

**Figure 1:**
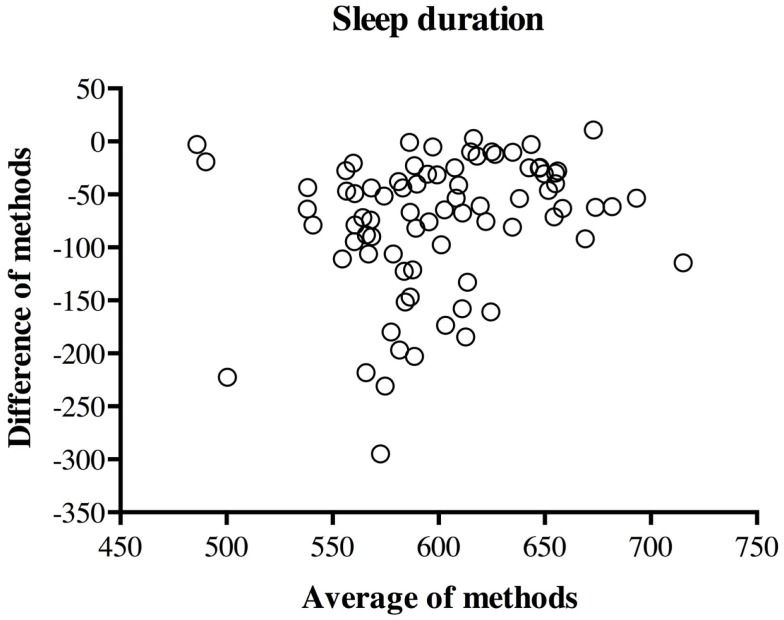
**Plot of between-methods difference by across-method average for sleep duration**.

The Bland–Altman plot of the difference (actigraphy–diary) against the mean [(actigraphy × diary)/2] is presented in Figure 1 for sleep duration. As can be seen from the plot, there was more variability in the between-methods difference for children who slept less. Moreover, nearly all mothers (97.5%) overestimated sleep duration when compared to actigraphy.

**Figure 2:**
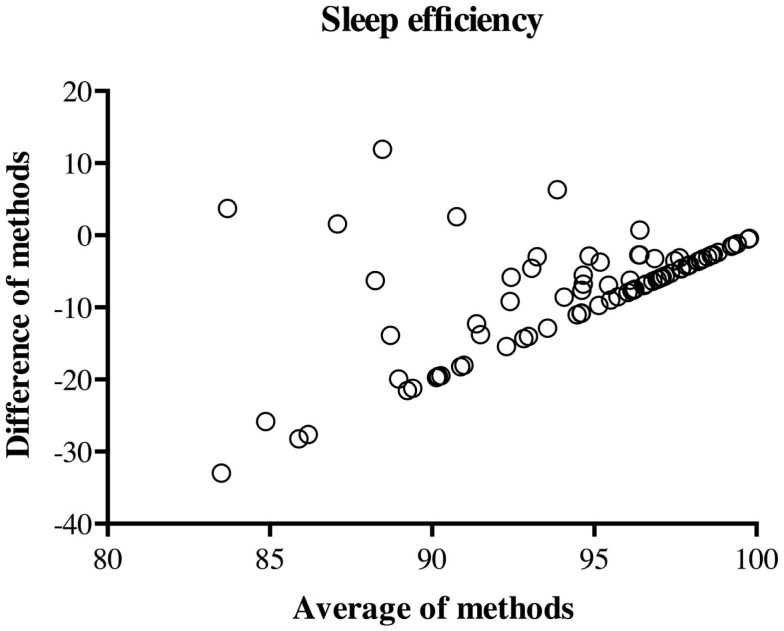
**Plot of between-methods difference by across-method average for sleep efficiency**.

The Bland–Altman plot of the difference against the mean for sleep efficiency is presented in Figure 2. As mentioned above, there was a linear relation between the difference and the mean of methods. More precisely, as sleep efficiency increases, the between-methods difference on sleep efficiency gets smaller, even more so than with sleep duration. Upon further investigation, this systematic bias appears to be caused by the fact that most mothers (63.8%) reported no awakening in the diary. In other words, since the majority of mothers reported that their children had perfect sleep efficiency (100%), only actigraphy-derived sleep efficiency varied in these cases, and thus, actigraphy accounted for both the average and the difference between the methods. In line with the findings concerning sleep duration, most mothers (92.5%) overestimated sleep efficiency in comparison to actigraphy.

### *Post hoc* analyses

The previous finding raises the question of whether the mothers who did report at least one awakening in their child in the diary differed from those who did not. *T-*tests revealed that mothers who reported at least one awakening in the diary perceived more sleep problems in their child as measured by the CBCL sleep problems scale [*M* = 3.96, SD = 2.83 vs. *M* = 2.57, SD = 2.46;*t*(69) = −2.16, *p* = 0.034]. Also, as expected, mothers who reported at least one awakening in the diary rated their child higher on the CBCL item “Wakes up often at night” [*M* = 0.72, SD = 0.74 versus *M* = 0.33, SD = 0.56; *t*(69) = −2.33, *p* = 0.025]. *T*-tests also revealed that actigraphic sleep duration and sleep efficiency did not differ between children whose mothers reported at least one awakening in the diary and children whose mothers did not.

## Discussion

The central aim of this study was to investigate the relations and level of agreement among actigraphy, a maternal sleep diary, and the parent-completed CBCL to assess sleep in toddlers. It was expected that good correspondence would be found between maternal and paternal CBCLs, between the mother-completed CBCL and the diary, and between actigraphy and the diary. Poor relations were expected between the CBCL completed by either parent and actigraphy. Hypotheses were generally confirmed, albeit with some noteworthy exceptions.

### Sleep diary and CBCL

First, the findings demonstrate that parents were generally consistent in the subjective evaluation of their child’s sleep quality. In addition to the correspondence between maternal and paternal CBCL scores, two of the three CBCL items that refer to and should impact sleep quality (“Nightmares,” “Wakes up often at night”), as well as the total sleep problems score, rated by both parents, were associated with mothers’ estimates of sleep efficiency in the diary. These results suggest that these CBCL items measure certain aspects of sleep that influence or are related to maternal perceptions of child sleep efficiency. Also, the parent’s ability to notice that the child is awake or having a nightmare relies on the child’s tendency to signal such events (14). It is to be expected, then, that part of the correspondence between these CBCL items and the sleep diary is attributable to a common underlying influence of child signaling tendencies, which both mothers and fathers might detect. Of course, analyses based on single items call for a certain amount of caution, given the inevitable greater measurement error as compared to multiple-item aggregate scores.

Second, results showed good correspondence between CBCL scores reported by mothers and maternal diaries regarding the evaluation of children’s sleep duration. Indeed, almost all of the CBCL items filled out by mothers that assess sleep problems that should further affect child sleep duration (“Does not want to sleep alone,” “Has trouble getting to sleep,” and “Resists going to bed at night”) were associated with mothers’ estimations of their child’s sleep duration in the diary. Because mothers are generally involved in a young child’s bedtime routine and are thus usually aware of when their child goes to sleep (19), these findings were expected. However, surprisingly, the item “Sleeps less than most children during day and/or night,” which is designed to assess sleep duration directly, was not correlated with mothers’ estimations of this same sleep variable in the diary. A similar finding was reported in a study conducted with older children regarding the agreement between the CBCL and PSG (9). Taken together, these results suggest that mothers’ subjective appreciation of what is normal sleep duration and how their own children compare to this (CBCL) is not related to children’s sleep duration when it is assessed objectively (actigraphy or PSG). It may be that many mothers are not familiar with average or expected sleep durations among children and thus are ill-equipped to respond to this CBCL item, which requires them to make normative comparisons.

Overall, correspondence between father-reported CBCL sleep scores and maternal diary-derived sleep duration (but not efficiency) was very poor, with no association reaching statistical significance. It has been demonstrated that mothers are more involved than fathers in their children’s bedtime routines (29), possibly resulting in less accurate perceptions among fathers. If fathers are less intensively involved in their children’s bedtime routines, they would indeed be less informed of their children’s actual sleep duration. However, this would not preclude them from being aware of their children’s night awakenings and thus lower sleep efficiency. In fact, the findings pertaining to the correspondence between the two parent-reported CBCLs support this hypothesis: higher correlations were found when considering sleep behaviors that should impact sleep efficiency than sleep behaviors that should impact sleep duration.

### Actigraphy and CBCL

Overall, results showed poor concordance between the CBCL filled out by either parent and sleep variables derived from actigraphy. Only mothers’ perceptions on two CBCL items (“Has trouble getting to sleep” and “Resists going to bed at night”) were associated with sleep duration as derived from actigraphy. These results are not surprising, given that these particular CBCL items reflect sleep onset as perceived by the mother, which, as reported in the diary, is used as a guide to determine actual sleep onset and thus to score actigraphy data. None of the other associations were statistically significant, including those involving the total sleep problems scale. This may appear somewhat surprising in light of data suggesting that parents of children with sleep problems experience lower-quality sleep themselves (34), suggesting that children’s sleep problems disrupt parents’ sleep, which should enhance parental awareness of their children’s sleep problems. It has also been observed that many infants with poor sleep quality are unable to self-soothe and to fall back asleep without parental intervention (35), which consequently should result in parents noticing their child’s sleep problems. It is, then, noteworthy that none of the associations between the CBCL and actigraphy-derived sleep efficiency, in particular, were significant. We see this overall pattern of very weak associations between the CBCL scores and sleep assessed objectively as quite important, given the frequent use of the CBCL as a cost-effective sleep measure. The current results suggest that this cost-effectiveness may be at the expense of accuracy, at least when aiming to estimate sleep duration or efficiency. The CBCL was developed to measure clinical problems and may be ill-suited to distinguish fine individual differences in sleep patterns in normative populations. In fact, a recent study (18) did illustrate that the gap between sleep estimated by actigraphy and by parental report (albeit not with the CBCL) is smaller in clinical groups. On the other hand, it is also possible that in some cases, parents detect or perceive sleep problems in their children who do not seem, objectively, to have such problems.

### Sleep diary and actigraphy

Consistent with previous studies, significant correlations were found between actigraphy- and diary-derived sleep duration, but not sleep efficiency [for a review, see Ref. (36)]. As mentioned above, the result concerning sleep duration is probably somewhat explained by the fact that sleep onset and sleep offset in actigraphy scoring were partly determined by sleep onset and sleep offset as recorded by mothers in diaries. Besides, the current study further suggests, with the use of the Bland and Altman (33) method, that agreement between actigraphy and the diary is, in fact, quite low, including on sleep duration despite the satisfactory rank-order correlation for this sleep variable. Even with the use of fairly lenient agreement criteria, there was satisfactory agreement for only about 70% of children. Similar findings have been reported by Werner et al. (20), who found poor agreement between diary- and actigraphy-derived nocturnal sleep and wake time, despite using more conservative agreement criteria than those used here. Several items might explain such poor agreement between measures. For example, it is possible that actigraphy and sleep diaries simply do not measure the same aspects of sleep, given that sleep diaries tap into a subjective perception of sleep, whereas actigraphy measures motor activity (20). Furthermore, as mentioned above, the parental diary’s ability to detect wakefulness in a child relies on the child’s tendency to signal awakenings or difficulties falling asleep – a tendency that not all young children show (37). Overall, the current study’s results converge with previous research by suggesting that a parental sleep diary does not provide a precise estimation of children’s sleep efficiency. Yet, sleep diaries may still be able to detect awakenings among children who signal such awakenings. When focusing on sleep duration, it appears that the appropriateness of a sleep diary depends on the intent. Given the correlations found between sleep diaries and actigraphy while controlling for confounding variables in the present and previous studies, the sleep diary (unlike the CBCL) may be a reasonable proxy to estimate children’s sleep duration for research purposes – that is, to link individual differences in sleep duration to individual differences in predictors or outcomes of interest. Also, the sleep diary may be a judicious choice when aiming to make within-child comparisons, such as in pre- and post-test study designs. However, in clinical settings where the interest should be to obtain a reasonably accurate estimate of specific individual children’s sleep minutes, or in studies aiming to provide descriptive statistics, such as average sleep times in the general or specific populations, actigraphy is a more appropriate choice. For instance, in this sample, even when allowing for a 2-h difference per night in estimates of sleep duration (which represents an extremely substantial difference), the sleep diary still fell short of providing satisfactory estimates of sleep duration for over 20% of the children. Therefore, when aiming to estimate individual children’s actual sleep duration and sleep efficiency, actigraphy is preferable, given its satisfactory agreement with PSG (12, 38–40). Finally, in light of finding a greater discrepancy in this study between actigraphy and diary estimates for children who sleep less or more poorly (Figures 1 and 2), particular caution is needed with children who experience lower-quality sleep.

### *Post hoc* analyses

The results of the *T*-tests revealed that “objective” sleep efficiency and duration are similar in children whose mothers did or did not report awakenings in their children in the diary, whereas some CBCL scores differed between these two groups. Altogether, these results suggest that mothers who report awakenings in their child in the diary also do on the CBCL but that this is not paralleled by more awakenings as measured by actigraphy. Consequently, these results support the above findings, which suggest that subjective sleep measures tend to converge with each other but not with a more objective measure of sleep such as actigraphy. These data also highlight the very low proportion of mothers who reported awakenings in the diary, which may point to a possible bias in the present study design pertaining to the 30-min window used for the sleep diary. It might be that some mothers did not understand that they were expected to note all awakenings, even those that lasted <30 min. However, according to actigraphy, 75% of the children in our sample awoke for more than 30 min during the night, suggesting the presence of a considerable between-methods gap in this sleep parameter. Unfortunately, since the ability of actigraphy to detect wakefulness in children has been criticized [e.g., Ref. (12, 30, 32)], we cannot really determine which measure, between actigraphy and sleep diaries, is a more accurate assessment of night awakenings.

### Limitations

This study presents limitations that need to be considered in interpreting the results. First, the modest sample size limited statistical power. The fact that most participants were college-educated and Caucasian also constitutes a limitation, in that findings may not generalize to samples characterized by greater economic, biological, or psychosocial risk. Additionally, the sleep diary recording of sleep–wake patterns in 30-min intervals versus the actigraphy recording in 30-s epochs necessarily limited the potential agreement between these two measures. Similarly, parents were asked to complete the CBCL according to their perception of their child’s sleep in the last two months, which also may have contributed to part of the observed differences between the CBCL and other sleep measures. In line with Kushnir and Sadeh (18), and due to the fact that the CBCL was created to measure clinical problems, it is possible that better correspondence between this questionnaire and other sleep measures would be found with clinical populations. Finally, although the agreement between subjective and objective sleep measures was evaluated in this study, an exciting avenue for future research lies in the use of polysomnographic sleep recordings to further investigate the degree of convergence between different sleep measures in toddlers.

## Conclusion

The present study is the first, to our knowledge, to examine the convergence among the CBCL, actigraphy, and sleep diaries with toddlers. Consistent with findings among older children groups, this study suggests that the CBCL sleep items, sleep diaries, and actigraphy tap into quite different aspects of sleep among toddlers. Choosing which of these sleep measures to use should be based on the exact aspects of sleep that one aims to assess. Overall, despite its frequent use, great care should be exercised before choosing the composite sleep score of the CBCL, given its very poor relations to objective sleep measures.

## Conflict of Interest Statement

The Review Editor Jean-Paul Praud declares that, despite being affiliated with the same institution as author Valérie Simard, the review process was handled objectively and no conflict of interest exists. The authors declare that the research was conducted in the absence of any commercial or financial relationships that could be construed as a potential conflict of interest.
